# Soluble EGFR, a hepatokine, and adipsin, an adipokine, are biomarkers correlated with distinct aspects of insulin resistance in type 2 diabetes subjects

**DOI:** 10.1186/s13098-020-00591-7

**Published:** 2020-09-29

**Authors:** Mayu Kyohara, Jun Shirakawa, Tomoko Okuyama, Yu Togashi, Ryota Inoue, Jinghe Li, Daisuke Miyashita, Yasuo Terauchi

**Affiliations:** 1 Department of Endocrinology and Metabolism, Graduate School of Medicine, Yokohama-City University, 3-9 Fukuura, Yokohama, 236-0004 Japan; 2 Laboratory of Diabetes and Metabolic Disorders, Institute for Molecular and Cellular Regulation (IMCR), Gunma University, 3-39-15 Showa-machi, Maebashi, 371-8512 Japan

**Keywords:** Insulin resistance, Metabolic syndrome, Type 2 diabetes

## Abstract

**Background:**

Insulin resistance can occur in all metabolic organs including the liver, adipose tissue, and skeletal muscles. Circulating soluble epidermal growth factor receptor (soluble EGFR) and adipsin levels are altered in obese diabetic mice and are possibly correlated with insulin resistance in both mice and humans. Here, we investigated the significance of soluble EGFR and adipsin as biomarkers for insulin resistance in Japanese subjects with type 2 diabetes.

**Methods:**

We measured the soluble EGFR and adipsin levels in sera from 47 non-diabetic subjects and 106 subjects with type 2 diabetes using enzyme-linked immunosorbent assays (ELISAs) and analyzed the correlations between the soluble EGFR or adipsin levels and metabolic parameters in type 2 diabetes subjects. We also measured the gene expression levels of *Egfr* and *Cfd* (adipsin) in the liver, adipose tissue, and skeletal muscle in mice with/without obesity or diabetes.

**Results:**

The soluble EGFR levels were correlated with the fasting blood glucose level (*P* = 0.010), HOMA-IR (*P* = 0.035), HbA1c level (*P* = 0.007), HDL-cholesterol level (*P* = 0.044), and FIB-4 index (*P* = 0.017) after adjustments for age, sex, and total cholesterol levels. These factors are known to be related to hepatic insulin resistance. The serum adipsin levels were correlated with BMI (*P* < 0.001), waist circumference (*P* < 0.001), fasting serum insulin level (*P* = 0.001), HOMA-IR (*P* = 0.009), CPR-index (*P* = 0.045), and FIB-4 index (*P* = 0.007) after adjustments for age, sex and eGFR levels. Abdominal adiposity leads to the potentiation of these factors. The expression of *Egfr* was abundant in the liver, while *Cfd* was predominantly expressed in adipose tissue in mice.

**Conclusions:**

Soluble EGFR, a hepatokine, is correlated with insulin resistance in the liver, while adipsin, an adipokine, is associated with adipose insulin resistance.

Trial registration: UMIN Clinical Trials Registry (www.umin.ac.jp), UMIN000020474. Registered 8 January 2016.

## Background

The growing number of patients with type 2 diabetes (T2DM) around the world is an issue that must be solved, but not all mechanisms of pathogenesis have been elucidated. A number of humoral factors are involved in the development and progression of diabetes through inter-organ crosstalk. To identify circulating factors involved in the pathogenesis of T2DM, we previously performed serum proteomic analyses in leptin receptor-mutated *db/db* mice [1]. The *db/db* mouse model is an animal model of T2DM with morbid obesity and insulin resistance that gradually develops diabetic complications. We conducted quantitative proteomic analyses using the following 2 protocols: (1) *db/db* mouse sera were compared with *db/*+ mouse sera obtained from animals at 4, 8, 12, and 24 weeks of age, and (2) *db/db* mouse sera from animals treated with liraglutide were compared with sera from animals without liraglutide treatment. We identified soluble epidermal growth factor receptor (soluble EGFR) and adipsin as common factors, since the protein levels of these factors changed significantly in sera from *db/db* mice or after treatment with liraglutide. We also reported the EGFR levels in sera from other diabetic mouse models as well as 15 subjects with normal glucose tolerance (NGT) and 27 patients with T2DM using an ELISA, and the results suggested that soluble EGFR is a potential biomarker for evaluating insulin resistance in both mice and humans [2].

EGFR, a transmembrane tyrosine kinase receptor, is a member of the ErbB family and is expressed on various cell types of epithelial, mesenchymal and neuronal origin [3]. Serum EGFR, which is known as a tumor marker for several cancers, is detected in healthy individuals or cancer patients as a soluble isoform that only contains the extracellular domain of the full-length EGFR [4]. Recently, Ji Min Kim et al. [5] reported that serum soluble EGFR levels were positively correlated with HbA1c and fasting glucose levels, but not with HOMA-IR, in patients who had been newly diagnosed as having T2DM.

Complement factor D (CFD), also known as adipsin, is a serine protease that is essential for alternative pathway activation [6] and is an adipokine secreted by adipocytes. Adipsin improves beta-cell function in mice and is decreased in the sera of T2DM patients with beta-cell failure [7]. Adipsin replenishment in *db/db* mice improves hyperglycemia and preserves the beta-cell mass by blocking dedifferentiation and death [8]. The serum adipsin levels in T2DM patients or in patients with insulin resistance remain controversial. Previous reports have shown that serum adipsin levels are lower in T2DM patients than in normal subjects [9–11], whereas they increase in subjects with T2DM as the severity of obesity increases [12]. Serum adipsin levels were also reported to be negatively correlated with HOMA-IR in T2DM patients [10, 11]. Patients with polycystic ovary syndrome (PCOS) who exhibited insulin resistance also had elevated serum adipsin levels, compared with control patients [13].

Insulin resistance is caused by the impairment of insulin receptor-mediated signaling in insulin-sensitive organs, i.e., the liver, adipose tissue, skeletal muscles, brain, and so on. Metabolic syndrome can be accompanied by fatty liver, visceral adiposity, muscular lipid accumulation (namely, ectopic fat accumulation), impaired insulin action via free fatty acids, micro-inflammation, and tissue-derived humoral factors (referred to as “adipokines” when they are from adipose tissue, “hepatokines” when they are from the liver, or “myokines” when they are from skeletal muscle). However, insulin resistance is often observed in subjects without obesity or lipodystrophy, especially in non-obese Asians [14].

In the present study, we focused on soluble EGFR and adipsin; these two factors were identified as candidate biomarkers correlated with insulin resistance in *db/db* mice, which exhibit severe obesity and hyperglycemia. However, the involvement of serum soluble EGFR and adipsin in insulin resistance remains unclear in the context of pathological conditions. Our previous findings showed that adipsin, an adipokine, might be correlated with fat insulin resistance in a manner that is independent of the aspect of insulin resistance that is correlated with soluble EGFR. To investigate the significance of soluble EGFR and adipsin as biomarkers for insulin resistance in Japanese subjects with T2DM, we measured the serum levels of EGFR and adipsin. We also confirmed the gene expressions of *Egfr* and *Cfd* (adipsin) in the liver, adipose tissue, and skeletal muscle of animal models. In this manner, we sought to reveal organ-specific biomarkers of insulin resistance in T2DM patients and to develop diagnostic strategies to optimize the treatment of diabetes.

## Materials and methods

### Ethics statement

This study was registered in the UMIN Clinical Trials Registry (UMIN000020474). All the subjects agreed to participate in this study by providing informed consent in accordance with the ethical committee regulations of the Yokohama City University Hospital (Approval no. B151201003).

### Patient selection

All the subjects, that were 106 T2DM patients and 47 NGT subjects with obesity and/or endocrine disease who had been admitted to Yokohama City University Hospital between January 2016 and March 2018, gave their written informed consent to participate in this study on admission. All the patients were between the ages of 20 and 70 years, inclusive. Patients with liver disease, renal dysfunction with a baseline estimated glomerular filtration rate (eGFR) of < 30 mL min^−1^ 1.73 m^−2^, severe ketosis or diabetic coma, severe infections, cancer, a perioperative status, or severe trauma were excluded from both the patient and the control groups in this study. The diagnosis of T2DM was based on the Report of the Committee of the Japan Diabetes Society on the Classification and Diagnostic Criteria of Diabetes Mellitus [15].

### Serum preparation

Whole blood was allowed to clot for 30 min at room temperature, then centrifuged at 2000×*g* for 10 min at 4 °C. The resulting supernatants were immediately stored as serum samples at − 80 °C until use.

### Clinical data and measurement of biochemical parameters

We measured the clinical data (age, sex, body mass index [BMI], waist circumference, systolic blood pressure, and diastolic blood pressure) on admission. The BMI was calculated as weight divided by the height squared (kg/m^2^). The height, weight, waist circumference, systolic blood pressure, and diastolic blood pressure were measured by medical staff according to standard procedures. Waist circumference was measured at the umbilical level while the subject was standing.

Blood samples were collected in the morning after an overnight fast on the 2nd day of admission. All the biochemical parameters, including fasting blood glucose (FBG), fasting serum insulin, C-peptide, HbA1c, total cholesterol (T-Chol), LDL-cholesterol (LDL-Chol), HDL-cholesterol (HDL-Chol), triglycerides (TG), AST, ALT, γGTP, and serum creatinine levels were concurrently measured using standard methods at the clinical laboratory of Yokohama City University Hospital. HbA1c was measured using high-performance liquid chromatography and was calculated according to the National Glycohemoglobin Standardization Program (NGSP). LDL-Chol was measured using a direct method. The eGFR was calculated as follows: eGFR (mL/min/1.73 m^2^) = 194 × serum creatinine^-1.094^ (mg/dL) × Age^-0.287^ (years) (for men), and eGFR (mL/min/1.73 m^2^) = 194 × serum creatinine^-1.094^ (mg/dL) × Age^-0.287^ (years) × 0.739 (for women) [16]. HOMA-IR, a marker of insulin resistance [17], was calculated as fasting insulin (μIU/mL) × fasting glucose (mg/dL)/405. The serum insulin and HOMA-IR data for T2DM patients treated with insulin were excluded from the analyses. The CPR-index, a marker of the insulin secretion capacity [18], was calculated as fasting CPR (ng/mL)/fasting glucose (mg/dL) × 100. The FIB‐4 index, a marker of hepatic fibrosis, was calculated as (age × AST [U/L])/(PLT [10^9^/L] × ALT [U/L]^1/2^) [19]. We measured the serum EGFR levels (DEGFR0; R&D Systems, Minneapolis, MN, USA) and serum adipsin levels (ELH-Adipsin; RayBiotech, Norcross, GA, USA) using enzyme-linked immunosorbent assays (ELISAs) according to the manufacturers’ instructions. The intra- and inter-assay coefficients of variation were 3.7%–5.5% and 9.4%–10.0% for EGFR and < 10% and < 12% for adipsin, respectively.

### Animals and animal care

Male BKS.Cg-Dock7^m^ +/+Lepr^db^/J (*db/db*) mice, B6.Cg-Lep^ob^/J (*ob/ob*) mice, and their controls (lean; *ob*/+ or +/+) were obtained from Charles River Japan (Yokohama, Japan). The *db/*+ and *db/db* mice were at 30 weeks of age. The *ob/ob* and their control lean mice were at 8 weeks of age. The male heterozygous pancreatic beta-cell-specific glucokinase knockout mice (β*Gck* +/− mice) [20], which were backcrossed with C57BL/6 J mice more than 10 times, were at 8 weeks of age. All the *db/db*, *ob/ob,* and β*Gck * +/− mice and their controls were fed normal chow. All the experiments were conducted using male littermates. All the animal procedures were performed in accordance with the NIH Principles of Laboratory Animal Care, institutional animal care guidelines, and the guidelines of the Animal Care Committee of the Yokohama City University (approval no. F-A-16-055). The animal housing rooms were maintained at a constant room temperature (25 °C) and with a 12-h light (7:00 AM), 12-h dark (7:00 PM) cycle.

### Biochemical parameters in mice

The plasma glucose levels and the serum insulin levels in mice were determined using Glutest Neo Super (Sanwa Kagaku Kenkyusho, Nagoya, Japan) and an insulin ELISA kit (M1102; Morinaga, Yokohama, Japan).

### Analysis of gene expression

Total RNAs from the liver and skeletal muscle tissues were isolated using a QIA shredder and an RNeasy kit (QIAGEN, Hilden, Germany). Total RNA from the epididymal fat was isolated using an RNeasy lipid tissue kit (QIAGEN). cDNA was prepared using High Capacity cDNA reverse transcription kits (Applied Biosystems, Foster City, CA, USA) and was subjected to quantitative PCR (7900 real-time PCR system; Applied Biosystems) using THUNDERBIRD SYBR qPCR Master Mix (Toyobo, Osaka, Japan). Each quantitative reaction was performed in duplicate. Data were normalized according to the β-actin level. The following primer sequences were used:

*Egfr* forward primer: 5′-GCCATCTGGGCCAAAGATACC-3′;

*Egfr* reverse primer: 5′-GTCTTCGCATGAATAGGCCAA-3′;

*Cfd* (*adipsin*) forward primer: 5′-CATGCTCGGCCCTACATGG-3′;

*Cfd* (*adipsin*) reverse primer: 5′-CACAGAGTCGTCATCCGTCAC-3′;

β-actin forward primer: 5′-CTAAGGCCAACCGTGAAAAGAT-3′;

β-actin reverse primer: 5′-CACAGCCTGGATGGCTACGT-3′.

### Statistical analysis

Data were tested for a normal distribution using the Anderson–Darling test. The data were reported as the mean ± SE, and data that were not normally distributed were reported as the medians [interquartile ranges] in Table 1. Statistical analyses using the Student t-test, Chi square test, and univariate and multivariate linear regression analyses were conducted with JMP statistical software (JMP Pro version 15.0.0; SAS Institute, Cary, NC, USA).

**Table 1:** Serum soluble EGFR and adipsin levels and metabolic characteristics of T2DM patients and NGT subjects

	NGT		T2DM		*P* value
	n	Value	n	Value	
Serum soluble EGFR (ng/mL)	47	62.3 ± 1.2	106	64.7 ± 0.9	0.11
Serum adipsin (μg/mL)	47	5.9 ± 0.3	106	5.8 [4.7–7.1]	0.55
Age (years)	47	54 [44–65]	106	56 [49–65]	0.06
Sex (No. men/women)	13/34	–	66/40	–	< 0.01
BMI	47	22.9 [20.6–29.4]	106	27.1 [23.9–30.9]	0.21
Waist circumference (cm)	25	90.0 [82.8–105.0]	71	96.0 [88.0–105.0]	0.33
Systolic blood pressure (mmHg)	42	126.5 [114.5–145.3]	102	134.4 ± 1.9	0.26
Diastolic blood pressure (mmHg)	42	82.2 ± 2.2	102	84.2 ± 1.3	0.4
Fasting blood glucose (mg/dL)	46	100.2 ± 1.3	106	161.0 [134.8–184.0]	< 0.01
Fasting serum insulin (μIU/mL)	41	8.2 [4.9–10]	83	9.6 [6.0–15.5]	0.16
C-peptide (ng/mL)	29	2.2 [1.4–3.2]	100	2.4 [1.6–3.2]	0.84
HOMA-IR	41	2.2 [1.1–2.7]	83	4.0 [2.4–6.4]	< 0.01
CPR-index	29	2.1 [1.5–2.9]	100	1.4 [0.9–2.1]	< 0.01
HbA1c (%)	45	5.7 ± 0.1	106	9.3 [7.9–10.2]	< 0.01
HbA1c (mmol/mol)	45	39 ± 0.5	106	78 [63–88]	< 0.01
Total cholesterol (mg/dL)	41	200 ± 5.9	102	187.0 [165.8–211.0]	0.24
LDL-cholesterol (mg/dL)	45	122.6 ± 4.6	105	110.0 [89.5–126.5]	0.03
HDL-cholesterol (mg/dL)	45	61.2 ± 2.6	106	42.0 [37.0–54.0]	< 0.01
Triglycerides (mg/dL)	45	100.0 [78.0–128.5]	106	141.5 [96–213.5]	< 0.01
AST (IU/L)	45	20.0 [15.5–22.5]	106	24.0 [18.0–38.0]	< 0.01
ALT (IU/L)	45	17.0 [11.0–25.5]	106	28.5 [19.0–50.0]	< 0.01
γGTP (IU/L)	44	21.5 [14.3–31.3]	105	37.0 [23.0–67.5]	0.04
FIB4-index	27	1.0 ± 0.1	95	1.6 ± 0.2	< 0.01
eGFR (mL/min/1.73 m^2^)	47	81.3 ± 2.5	106	83.2 ± 2.0	0.58

BMI, Body mass index; EGFR, Epidermal growth factor receptor; eGFR, Estimated glomerular filtration rate; NGT, Normal glucose tolerance; T2DM, Type 2 diabetes

The univariate linear regression analyses shown in Table 2 were performed using soluble EGFR as a dependent variable and all the other variables shown in Table 1 as independent variables. The multivariate linear regression analyses shown in Table 3 were performed using the soluble EGFR level as a dependent variable and age, sex, and all the other variables shown in Table 2 as independent variables (model 1) or age, sex, T-Chol level, and all the variables that exhibited a significant correlation in model 1 (FBG, HOMA-IR, HbA1c, HDL-Chol, TG and FIB-4 index) as independent variables (model 2). The univariate linear regression analyses shown in Table 4 were performed using adipsin as a dependent variable and all the other variables shown in Table 1 as independent variables. The multivariate linear regression analyses in Table 5 were performed using the adipsin level as a dependent variable and age, sex and all the other variables shown in Table 4 as independent variables (model 1) or age, sex, eGFR level, and all the variables that exhibited a significant correlation in model 1 (BMI, waist circumference, fasting serum insulin, HOMA-IR, CPR-index, and FIB-4 index) as independent variables (model 2). Variables that were not normally distributed were log-transformed for both the univariate and multivariate linear regression analyses. The raw data for several variables (age for both NGT subjects and T2DM patients, C-peptide for T2DM patients, and CPR-index for T2DM patients) were roughly normally distributed but did not yield normal distributions after log-transformation because of increased kurtosis and skewness. Consequently, we analyzed these variables as raw data in the univariate and multivariate linear regression analyses. Significance was defined as a *P* value of < 0.05 (*) or < 0.01 (**).

**Table 2:** Univariate linear regression analyses for serum soluble EGFR levels in T2DM patients and NGT subjects

	NGT			T2DM		
	n	β	*P*	n	β	*P*
Serum adipsin (μg/mL)	47	0.035	0.815	106	0.095	0.332
Age (years)	47	0.093	0.533	106	− 0.398	< 0.001
Sex	47	0.050	0.740	106	− 0.005	0.960
BMI	47	0.155	0.299	106	0.252	0.009
Waist circumference (cm)	25	− 0.044	0.833	71	0.242	0.042
Systolic blood pressure (mmHg)	42	− 0.029	0.856	102	− 0.143	0.152
Diastolic blood pressure (mmHg)	42	− 0.022	0.891	102	0.064	0.523
Fasting blood glucose (mg/dL)	46	0.164	0.275	106	0.285	0.003
Fasting serum insulin (μIU/mL)	41	0.219	0.169	83	0.233	0.034
C-peptide (ng/mL)	29	0.087	0.654	100	0.250	0.012
HOMA-IR	41	0.229	0.151	83	0.317	0.004
CPR-index	29	0.085	0.662	100	0.135	0.180
HbA1c (%, mmol/mol)	45	0.212	0.152	106	0.364	< 0.001
Total cholesterol (mg/dL)	41	0.361	0.021	102	0.419	< 0.001
LDL-cholesterol (mg/dL)	45	0.030	0.845	105	0.201	0.040
HDL-cholesterol (mg/dL)	45	0.064	0.675	106	0.085	0.386
Triglycerides (mg/dL)	45	0.466	0.112	106	0.309	0.001
AST (IU/L)	45	0.309	0.039	106	0.130	0.184
ALT (IU/L)	45	0.214	0.158	106	0.113	0.247
γGTP (IU/L)	44	0.124	0.423	105	0.213	0.029
FIB4-index	27	− 0.102	0.614	95	0.050	0.628
eGFR (mL/min/1.73 m^2^)	47	− 0.342	0.018	106	0.135	0.168

BMI, Body mass index; EGFR, Epidermal growth factor receptor; eGFR, Estimated glomerular filtration rate; NGT, Normal glucose tolerance; T2DM, Type 2 diabetes

**Table 3:** Multivariate linear regression analyses for serum soluble EGFR levels in T2DM patients

	Model 1			Model 2		
	n	β	*P*	n	β	*P*
Serum adipsin (μg/mL)	106	0.100	0.273			
BMI	106	0.070	0.502			
Waist circumference (cm)	71	0.072	0.551			
Systolic blood pressure (mmHg)	102	− 0.105	0.247			
Diastolic blood pressure (mmHg)	102	− 0.010	0.918			
Fasting blood glucose (mg/dL)	106	0.281	0.002	106	0.221	0.010
Fasting serum insulin (μIU/mL)	83	0.133	0.203			
C-peptide (ng/mL)	100	0.129	0.181			
HOMA-IR	83	0.224	0.030	83	0.193	0.035
CPR-index	100	− 0.015	0.873			
HbA1c (%, mmol/mol)	106	0.342	< 0.001	106	0.240	0.007
Total cholesterol (mg/dL)	102	0.314	< 0.001	–	–	–
LDL-cholesterol (mg/dL)	105	0.145	0.114			
HDL-cholesterol (mg/dL)	106	0.189	0.047	106	0.182	0.044
Triglycerides (mg/dL)	106	0.198	0.041	106	0.014	0.894
AST (IU/L)	106	0.089	0.337			
ALT (IU/L)	106	0.020	0.836			
γGTP (IU/L)	105	0.173	0.064			
FIB4-index	95	0.199	0.042	95	0.222	0.017
eGFR (mL/min/1.73 m^2^)	106	− 0.024	0.804			

Model 1: Adjusted for age and sex

Model 2: Adjusted for age, sex, and total cholesterol levels

BMI, Body mass index; EGFR, Epidermal growth factor receptor; eGFR, Estimated glomerular filtration rate; T2DM, Type 2 diabetes

**Table 4:** Univariate linear regression analyses for serum adipsin levels in T2DM patients and NGT subjects

	NGT			T2DM		
	n	β	*P*	n	β	*P*
Serum soluble EGFR (ng/mL)	47	0.035	0.815	106	0.095	0.332
Age (years)	47	0.021	0.887	106	− 0.039	0.693
Sex	47	− 0.147	0.323	106	− 0.162	0.098
BMI	47	0.520	< 0.001	106	0.420	< 0.001
Waist circumference (cm)	25	0.620	< 0.001	71	0.430	< 0.001
Systolic blood pressure (mmHg)	42	− 0.023	0.885	102	0.066	0.508
Diastolic blood pressure (mmHg)	42	0.080	0.613	102	0.098	0.328
Fasting blood glucose (mg/dL)	46	0.057	0.709	106	− 0.114	0.244
Fasting serum insulin (μIU/mL)	41	0.195	0.223	83	0.365	< 0.001
C-peptide (ng/mL)	29	0.304	0.109	100	0.157	0.119
HOMA-IR	41	0.194	0.225	83	0.300	0.006
CPR-index	29	0.324	0.086	100	0.184	0.067
HbA1c (%, mmol/mol)	45	− 0.012	0.940	106	0.058	0.553
Total cholesterol (mg/dL)	41	− 0.123	0.445	102	− 0.002	0.983
LDL-cholesterol (mg/dL)	45	− 0.011	0.943	105	0.141	0.150
HDL-cholesterol (mg/dL)	45	− 0.315	0.035	106	− 0.071	0.468
Triglycerides (mg/dL)	45	0.088	0.565	106	0.009	0.930
AST (IU/L)	45	0.157	0.303	106	0.133	0.174
ALT (IU/L)	45	0.187	0.219	106	0.133	0.173
γGTP (IU/L)	44	0.079	0.611	105	0.158	0.107
FIB4-index	27	− 0.018	0.931	95	0.226	0.028
eGFR (mL/min/1.73 m^2^)	47	− 0.365	0.012	106	− 0.339	< 0.001

BMI, Body mass index; EGFR, Epidermal growth factor receptor; eGFR, Estimated glomerular filtration rate; NGT, Normal glucose tolerance; T2DM, Type 2 diabetes

**Table 5:** Multivariate linear regression analyses for serum adipsin levels in T2DM patients

	Model 1			Model 2		
	n	β	*P*	n	β	*P*
Serum soluble EGFR (ng/mL)	106	0.117	0.273			
BMI	106	0.541	< 0.001	106	0.470	< 0.001
Waist circumference (cm)	71	0.523	< 0.001	71	0.451	< 0.001
Systolic blood pressure (mmHg)	102	0.045	0.653			
Diastolic blood pressure (mmHg)	102	0.060	0.565			
Fasting blood glucose (mg/dL)	106	− 0.106	0.280			
Fasting serum insulin (μIU/mL)	83	0.381	< 0.001	83	0.345	0.001
C-peptide (ng/mL)	100	0.187	0.076			
HOMA-IR	83	0.312	0.006	83	0.281	0.009
CPR-index	100	0.224	0.031	100	0.199	0.045
HbA1c (%, mmol/mol)	106	0.044	0.659			
Total cholesterol (mg/dL)	102	0.001	0.992			
LDL-cholesterol (mg/dL)	105	− 0.133	0.178			
HDL-cholesterol (mg/dL)	106	− 0.035	0.736			
Triglycerides (mg/dL)	106	− 0.019	0.859			
AST (IU/L)	106	0.112	0.261			
ALT (IU/L)	106	0.106	0.308			
γGTP (IU/L)	105	0.133	0.188			
FIB4-index	95	0.214	0.045	95	0.277	0.007
eGFR (mL/min/1.73 m^2^)	106	− 0.380	< 0.001	–	–	–

Model 1: Adjusted for age and sex

Model 2: Adjusted for age, sex, and eGFR levels

BMI, Body mass index; EGFR, Epidermal growth factor receptor; eGFR, Estimated glomerular filtration rate; T2DM, Type 2 diabetes

## Results

### Serum soluble EGFR and adipsin levels and metabolic characteristics of T2DM patients and NGT subjects

We determined the serum soluble EGFR and adipsin levels in sera from 47 NGT subjects and 106 T2DM patients using ELISAs (Table 1). The T2DM group had more male subjects than the NGT group. The T2DM patients had significantly higher FBG levels (*P* < 0.01), HOMA-IR levels (*P* < 0.01), and HbA1c levels (*P* < 0.01) and a lower CPR-index (*P* < 0.01), which is a marker of the insulin secretion capacity [18], compared with the NGT subjects. Significantly higher TG levels (*P* < 0.01), AST levels (*P* < 0.01), ALT levels (*P* < 0.01), γGTP levels (*P* = 0.04), and FIB-4 index (*P* < 0.01) and lower LDL-Chol levels (*P* = 0.03) were also observed in the T2DM patients. No differences in BMI, waist circumference, or renal function were observed between the T2DM group and the NGT group (Table 1).

### Univariate linear regression analyses for serum soluble EGFR levels in T2DM patients and NGT subjects

The correlations between the serum soluble EGFR levels and metabolic parameters in T2DM patients and NGT subjects were assessed using univariate linear regression analyses. No correlations were observed between the soluble EGFR and adipsin levels in sera from either T2DM patients or NGT subjects (Table 2). Among the T2DM patients, the serum soluble EGFR level was positively correlated with age (*P* < 0.001), BMI (*P* = 0.009), waist circumference (*P* = 0.042), and FBG level (*P* = 0.003), fasting serum insulin level (*P* = 0.034), C-peptide level (*P* = 0.012), HOMA-IR (*P* = 0.004), HbA1c level (*P* < 0.001), T-Chol level (*P* < 0.001), LDL-Chol level (*P* = 0.040), TG level (*P* = 0.001), and γGTP level (*P* = 0.029) (Table 2). Among the NGT subjects, the serum soluble EGFR level was positively correlated with the T-Chol level (*P* = 0.021) and the AST level (*P* = 0.039) and negatively correlated with the eGFR (*P* = 0.018) (Table 2).

### Multivariate linear regression analyses for serum soluble EGFR levels in T2DM patients

Among the T2DM patients, after adjustments for age and sex (Model 1), the serum soluble EGFR levels were positively correlated with the FBG level (*P* = 0.002), HOMA-IR (*P* = 0.030), HbA1c level (*P* < 0.001), T-Chol level (*P* < 0.001), HDL-Chol level (*P* = 0.047), TG level (*P* = 0.041) and the FIB-4 index (*P* = 0.042) (Table 3). Because the serum soluble EGFR levels were strongly correlated with the serum T-Chol levels, we also analyzed the correlations between the serum soluble EGFR level and parameters after adjustments for the T-Chol level as well as for age and sex. After adjustments for age, sex, and T-Chol levels (Model 2), the serum soluble EGFR levels were positively correlated with the FBG level (*P* = 0.010), HOMA-IR (*P* = 0.035), HbA1c level (*P* = 0.007), HDL-Chol level (*P* = 0.044) and the FIB-4 index (*P* = 0.017) (Table 3).

### Univariate linear regression analyses for serum adipsin levels in T2DM patients and NGT subjects

In the T2DM patients, the serum adipsin level was positively correlated with BMI (*P* < 0.001), waist circumference (*P* < 0.001), fasting serum insulin (*P* < 0.001), HOMA-IR (*P* = 0.006), and the FIB-4 index (*P* = 0.028) and negatively correlated with eGFR (*P* = < 0.001) (Table 4). The serum adipsin level was positively correlated with BMI (*P* < 0.001) and waist circumference (*P* < 0.001) and negatively correlated with the HDL-Chol level (*P* = 0.035) and the eGFR (*P* = 0.012) in the NGT subjects (Table 4).

### Multivariate linear regression analyses for serum adipsin levels in T2DM patients

In the T2DM patients, after adjustments for age and sex (Model 1), the serum adipsin levels were positively correlated with BMI (*P* < 0.001), waist circumference (*P* < 0.001), fasting serum insulin (*P* < 0.001), HOMA-IR (*P* = 0.006), CPR-index (*P* = 0.031), and the FIB-4 index (*P* = 0.045), and were negatively correlated with eGFR (*P* < 0.001) (Table 5). Furthermore, because the serum adipsin levels were strongly correlated with the eGFR levels, we also analyzed the correlations between the serum adipsin level and parameters after adjustments for the eGFR level as well as for age and sex. After adjustments for age, sex and the eGFR levels (Model 2), the serum adipsin level was still positively correlated with BMI (*P* < 0.001), waist circumference (*P* = 0.001), fasting serum insulin (*P* = 0.001), HOMA-IR (*P* = 0.009), CPR-index (*P* = 0.045), and the FIB-4 index (β = 0.327, *P* = 0.007) (Table 5).

### EGFR and adipsin expression levels in insulin-sensitive organs from diabetic mice

**Fig. 1:**
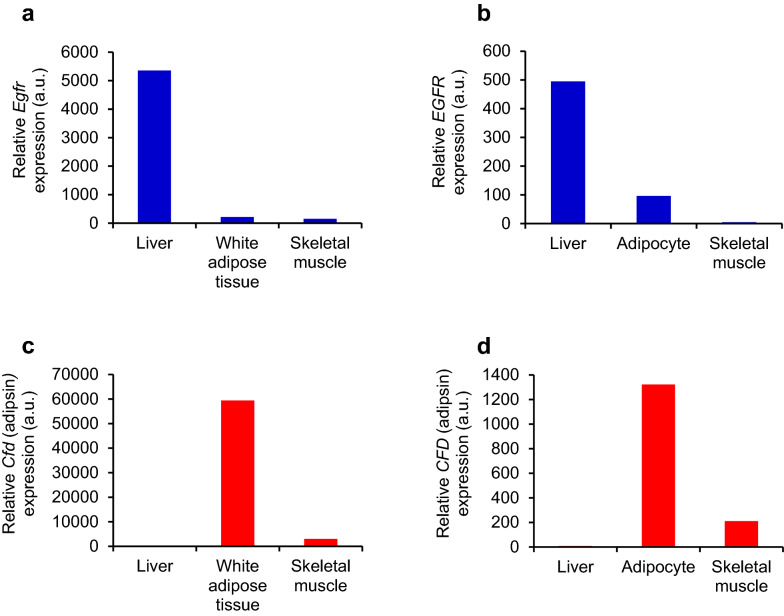
Tissue EGFR and adipsin levels in insulin-sensitive organs in mice and humans. **a**–**b** Relative gene expression levels of *EGFR* in the liver, adipose tissue, and skeletal muscle of mice (**a**) and humans (**b**). **c**–**d** Relative gene expression levels of *CFD* (adipsin) in the liver, adipose tissue, and skeletal muscle of mice (**c**) and humans (**d**). These findings were adapted from the BioGPS database (http://biogps.org)

Based on the BioGPS database (http://biogps.org/) [21], *EGFR* is abundantly expressed in the liver in both mice (Fig. 1a) and humans (Fig. 1b), compared with adipose tissue or skeletal muscle. Meanwhile, *CFD* (*adipsin*) is predominantly expressed in adipose tissue in both mice and humans (Fig. 1c, d). These results suggest that serum-circulating soluble EGFR and adipsin are derived from the liver and adipose tissue as a hepatokine and an adipokine, respectively.

**Fig. 2:**
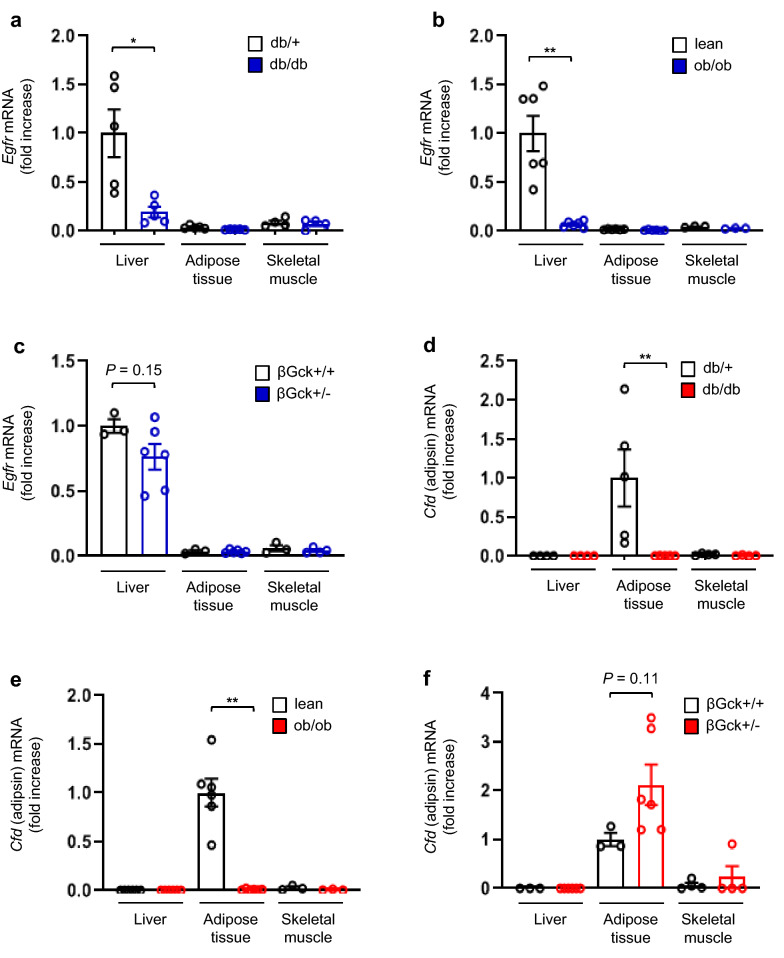
Tissue EGFR and adipsin levels in insulin-sensitive organs from *db/db, ob/ob,* and β*Gck * +/− mice. **a**–**c** *Egfr* mRNA expression levels in the liver, adipose tissue, and skeletal muscle of *db/*+ and *db/db* mice at 30 weeks of age, control lean and *ob/ob* mice at 8 weeks of age, and β*Gck* +/− and β*Gck* +/+ mice at 8 weeks of age. **d**–**f** *Cfd* (adipsin) mRNA expression levels in the liver, adipose tissue, and skeletal muscle of *db/*+ and *db/db* mice at 30 weeks of age, control lean and *ob/ob* mice at 8 weeks of age, and β*Gck* +/− and β*Gck *+/+ mice at 8 weeks of age. *P < 0.05, **P < 0.01 (n = 3–6 per group)

We next measured the gene expression levels of *Egfr* and *Cfd* (*adipsin*) in the liver, adipose tissue, and skeletal muscle in mice with/without obesity or diabetes. In *db/db* mice, the mRNA expression levels of *Egfr* were much higher in the liver than in adipose tissue or skeletal muscle. Furthermore, in the liver, the mRNA expression levels of *Egfr* were significantly lower in the *db/db* mice than in the *db/*+ mice (Fig. 2a). In *ob/ob* mice, which are also a model of obesity and insulin resistance similar to the *db/db* mouse model, the mRNA expression levels of *Egfr* were also much higher in the liver than in adipose tissue or skeletal muscle. In the liver, the mRNA expression levels of *Egfr* were also significantly lower in the *ob/ob* mice than in control lean mice (Fig. 2b). In heterozygous pancreatic beta-cell-specific glucokinase knockout mice (β*Gck * +/− mice), which exhibit impaired insulin secretion in response to glucose without insulin resistance, the mRNA expression levels of *Egfr* were also much higher in liver than in adipose tissue and skeletal muscle. Unlike *db/db* and *ob/ob* mice, no differences in the mRNA expression levels of *Egfr* in the liver were seen between the control and β*Gck * +/− mice (Fig. 2c).

On the other hand, the mRNA expression of *Cfd* (*adipsin*) was predominantly observed in the adipose tissue, rather than in the liver or skeletal muscle, in *db/db* mice. In the adipose tissue, the mRNA expression of *Cfd* was significantly lower in the *db/db* mice than in the *db/*+ mice (Fig. 2d). In *ob/ob* mice, the mRNA expression of *Cfd* was also much higher in the adipose tissue than in the liver or skeletal muscle. In the adipose tissue, the mRNA expression of *Cfd* was also significantly lower in the *ob/ob* mice than in the lean mice (Fig. 2e). In β*Gck* +/− mice, the mRNA expression of *Cfd* was also much higher in the adipose tissue than in the liver and skeletal muscle. Unlike the *db/db* and *ob/ob* mice, no difference in the mRNA expression of *Cfd* in adipose tissue was seen between the control and β*Gck *+/− mice (Fig. 2f).

## Discussion

In this study, we examined the circulating levels of soluble EGFR and adipsin in sera from normal and T2DM human subjects. The serum soluble EGFR levels in T2DM patients were positively correlated with the FBG, HOMA-IR, HbA1c, T-Chol, HDL-Chol, and FIB-4 index. Meanwhile, the serum adipsin levels in T2DM patients were positively correlated with BMI, waist circumference, fasting serum insulin, HOMA-IR, CPR-index, and the FIB-4 index and negatively correlated with the eGFR.

**Fig. 3:**
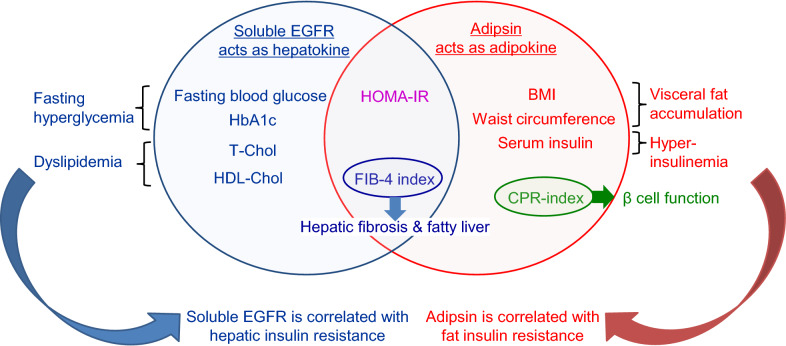
Metabolic parameters correlated with serum soluble EGFR and adipsin levels in T2DM patients. The Venn diagram shows the metabolic parameters correlated with serum soluble EGFR and adipsin levels in the T2DM patients

When these parameters were categorized according to the correlation with soluble EGFR or adipsin, distinctive features of insulin resistance emerged (Fig. 3). Because hepatic insulin signaling regulates the activity of transcription factors involved in gluconeogenesis, glycolysis, and fatty acid synthesis [22–24], insulin resistance in the liver increases glucose production in the liver, resulting in fasting hyperglycemia or lipoprotein production. The correlation of soluble EGFR with hyperlipidemia and fasting hyperglycemia is associated with the correlation between soluble EGFR and hepatic insulin resistance. Visceral adipose tissue is considered to have a stronger contribution to insulin resistance than subcutaneous adipose tissue [25]. Since adipose tissue is one of the largest target organs of insulin, adipose insulin resistance because of visceral obesity contributes to systemic insulin resistance and consequent hyperinsulinemia [26]. The measurement of waist circumference is important for predicting the volume of visceral fat. Visceral adiposity could result in the abnormal production of adipokines, i.e., adiponectin, leptin, or proinflammatory cytokines [27]. The association of adipsin with BMI, waist circumference, and hyperinsulinemia suggests that adipsin is correlated with adipose tissue insulin resistance.

A clear tendency toward the tissue-specific expression of EGFR and adipsin was observed in the liver, adipose tissue, and skeletal muscle in mice and humans. *Egfr* expression levels were much higher in the liver but were reduced in the livers of *db/db* and *ob/ob* mice, which exhibit insulin resistance. The EGFR expression levels were also reduced in sera from these mouse models, as shown in our previous report [2]. Meanwhile, the expression levels of *Cfd* (adipsin), an adipokine, were higher in adipose tissue and were reduced in adipose tissues from *db/db* and *ob/ob* mice. The adipsin expressions were also reduced in sera from these mice [2]. These results also support the idea that soluble EGFR is a hepatokine that is mainly correlated with insulin resistance in the liver, while adipsin is an adipokine that is mainly associated with insulin resistance in adipose tissue. Several molecules have been reported as hepatokines and adipokines involved in insulin resistance, such as selenoprotein P [28] and adiponectin [29], respectively. Further studies on the relationships among these hepatokines and adipokines with soluble EGFR or adipsin are warranted.

Consistent with our results, Ji Min Kim et al. [5] reported that the serum soluble EGFR levels were positively correlated with the FBG, HbA1c, T-Chol, LDL-Chol, and TG levels in patients who had been newly diagnosed as having T2DM. No correlation was observed between soluble EGFR and the HOMA-IR or serum insulin levels in their study. We reported that the soluble EGFR levels were significantly correlated with the HOMA-IR levels, but not with the serum insulin levels. This positive correlation with HOMA-IR seemed to be due to hyperglycemia, rather than hyperinsulinemia. As for adipsin, consistent with our study, Vasilenko et al. [12] reported that the serum adipsin levels increased in parallel with an increasing degree of obesity in both patients with and those without T2DM. Jun-Sing Wang et al. [11] reported that the serum adipsin levels were negatively associated with the HOMA-IR levels in NGT subjects and patients with IGT or T2DM after adjustments for age, sex, and BMI. In our study, the correlation between the serum adipsin and HOMA-IR levels vanished after adjustments for BMI in addition to those for age and sex (data not shown). This finding supports the hypothesis that the serum adipsin levels are more strongly correlated with insulin resistance associated with obesity because of the expansion of visceral fat. The subjects in the above-mentioned study had relatively lower BMIs than those in our study, which might have affected this difference in the correlation between serum adipsin and HOMA-IR levels. HOMA-IR is calculated by multiplying the fasting glucose level with the fasting insulin level. Thus, the correlation between serum adipsin and the HOMA-IR levels in that study might have been influenced by the blood glucose levels, rather than the insulin levels. Recently, Nicolás Gómez-Banoy et al. [8] reported that adipsin preserved the beta-cells in *db/db* mice, and higher concentrations of serum adipsin were associated with a significantly lower risk of developing future diabetes in humans. Several reports have shown that the serum adipsin levels were decreased in T2DM patients [9–11]. The serum adipsin levels might be associated with not only fat insulin resistance, but also potential beta cell function, which is consistent with our result that the serum adipsin level was positively correlated with the CPR-index, a marker of the insulin secretion capacity in T2DM patients (Fig. 3). Further studies involving a larger number of subjects are required to clarify the correlation between the serum adipsin level and beta cell function or fat insulin resistance.

The serum adipsin levels were also positively correlated with the FIB-4 index, a marker of hepatic fibrosis, in T2DM patients (Fig. 3). Qiu et al. [30] reported that patients with non-alcoholic fatty liver disease (NAFLD) had higher serum adipsin levels than control subjects. Meanwhile, Yusuf Yilmaz et al. [31] showed that the serum adipsin levels did not differ between biopsy-proven NAFLD patients and controls and were not correlated with liver fibrosis. Thus, the serum adipsin levels in NAFLD patients remain controversial. Fatty liver is caused by insulin resistance in either liver or adipose tissue, as demonstrated by the fact that adipocyte-specific insulin receptor knockout (FIRKO) mice develop hepatic steatosis [32]. Therefore, our result showing an association between adipsin and the FIB-4 index also supports the hypothesis that adipsin is a marker of fat insulin resistance in T2DM patients.

We showed significant correlations between the serum soluble EGFR level and liver insulin resistance and between the serum adipsin level and fat insulin resistance in this study. However, no differences in serum soluble EGFR and adipsin levels were seen between the T2DM patients and the NGT subjects. The reason for this might be due to the small sample size. Furthermore, remarkable obesity, a cause of insulin resistance, is rare among both T2DM patients and NGT subjects in the Japanese population; this may obscure differences in the serum soluble EGFR and adipsin levels between these two groups.

In the current study, the serum adipsin levels were negatively correlated with the eGFR levels. The serum adipsin levels are regulated through catabolism in the kidney, where adipsin is filtered by the glomerulus and reabsorbed by the proximal tubule [33]. The serum adipsin levels are reportedly elevated in patients with renal failure [33].

In conclusion, we demonstrated that a hepatokine, soluble EGFR, and an adipokine, adipsin, were correlated with distinct aspects of insulin resistance in human T2DM subjects, suggesting that soluble EGFR can be used as a biomarker for hepatic insulin resistance and hyperglycemia and that adipsin can be used as a biomarker for fat insulin resistance and visceral fat accumulation. In this study, we newly revealed two candidates for organ-specific biomarkers of insulin resistance in T2DM patients; these two biomarkers could be of potential use in the development of diagnosis methods and therapeutic strategies for diabetes.

## Data Availability

All data analyzed during this study are included in this manuscript.

## Abbreviations

BMI: Body mass index
CFD: Complement factor D
EGFR: Epidermal growth factor receptor
eGFR: Estimated glomerular filtration rate
ELISA: Enzyme-linked immunosorbent assay
HDL-Chol: HDL-cholesterol
LDL-Chol: LDL-cholesterol
NGT: Normal glucose tolerance
T-Chol: Total cholesterol
TG: Triglycerides
T2DM: Type 2 diabetes
